# Bis(2,2′-bi-1*H*-imidazole-κ^2^ *N* ^3^,*N* ^3′^)(thio­cyanato-κ*N*)copper(II) chloride

**DOI:** 10.1107/S1600536809044717

**Published:** 2009-10-31

**Authors:** Qi Ma, Shuang-Ming Meng, Feng Feng, Li-Ping Lu, Miao-Li Zhu

**Affiliations:** a College of Chemistry and Chemical Engineering, Shanxi Datong University, Datong, Shanxi 037009, People’s Republic of China; b Institute of Molecular Science, Key Laboratory of Chemical Biology and Molecular Engineering of the Education Ministry, Shanxi University, Taiyuan, Shanxi 030006, People’s Republic of China

## Abstract

In the title salt, [Cu(NCS)(C_6_H_6_N_4_)_2_]Cl, the Cu^II^ atom adopts a five-coordinated square-pyramidal geometry consisting of an N atom from a thio­cyanate anion and four N atoms from two chealting biimidazole ligands. The thio­cyanate ligand occupies the axial position and is, like the Cu^II^ centre, located on a mirror plane. The cation and anion are linked into a linear chain by N—H⋯S and N—H⋯Cl hydrogen bonds.

## Related literature

For the neutral mol­ecule 2,2′-biimidazole (H_2_biim) and its monoanionic derivative (Hbiim^−^), see: Tadokoro & Nakasuji (2000 ▶). Thio­cyanate is a versatile bridging ligand, see: Ribas *et al.* (1998 ▶). For Cu—N bond lengths in biimidazole–Cu complexes, see: Govor *et al.* (2008 ▶);
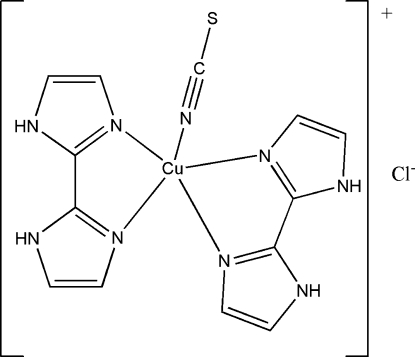


## Experimental

### 

#### Crystal data

[Cu(NCS)(C_6_H_6_N_4_)_2_]Cl
*M* *_r_* = 425.37
Orthorhombic, 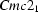

*a* = 12.900 (5) Å
*b* = 9.442 (4) Å
*c* = 12.888 (5) Å
*V* = 1569.8 (11) Å^3^
*Z* = 4
Mo *K*α radiation
μ = 1.71 mm^−1^
*T* = 298 K
0.10 × 0.10 × 0.10 mm

#### Data collection

Bruker SMART CCD area-detector diffractometer
Absorption correction: multi-scan (*SADABS*; Sheldrick, 1996 ▶) *T*_min_ = 0.610, *T*_max_ = 0.847
3518 measured reflections
1291 independent reflections
1254 reflections with *I* > 2σ(*I*)
*R*_int_ = 0.046

#### Refinement

*R*[*F* ^2^ > 2σ(*F* ^2^)] = 0.047
*wR*(*F* ^2^) = 0.096
*S* = 1.20
1291 reflections
121 parameters
1 restraint
H-atom parameters constrained
Δρ_max_ = 0.45 e Å^−3^
Δρ_min_ = −0.45 e Å^−3^
Absolute structure: Flack (1983 ▶), 539 Friedel pairs
Flack parameter: 0.06 (3)

### 

Data collection: *SMART* (Bruker, 2000 ▶); cell refinement: *SAINT* (Bruker, 2000 ▶); data reduction: *SAINT*; program(s) used to solve structure: *SHELXS97* (Sheldrick, 2008 ▶); program(s) used to refine structure: *SHELXL97* (Sheldrick, 2008 ▶); molecular graphics: *SHELXTL/PC* (Sheldrick, 2008 ▶); software used to prepare material for publication: *publCIF* (Westrip, 2009 ▶).

## Supplementary Material

Crystal structure: contains datablocks I, global. DOI: 10.1107/S1600536809044717/ng2677sup1.cif

Structure factors: contains datablocks I. DOI: 10.1107/S1600536809044717/ng2677Isup2.hkl

Additional supplementary materials:  crystallographic information; 3D view; checkCIF report

## Figures and Tables

**Table 1:** Selected bond lengths (Å)

Cu1—N1	2.014 (6)
Cu1—N3	2.031 (5)
Cu1—N5	2.344 (10)

**Table 2:** Hydrogen-bond geometry (Å, °)

*D*—H⋯*A*	*D*—H	H⋯*A*	*D*⋯*A*	*D*—H⋯*A*
N2—H2*A*⋯Cl1^ii^	0.86	2.27	3.092 (5)	160
N4—H4⋯Cl1	0.86	2.52	3.305 (6)	153
N2—H2*A*⋯S1^iii^	0.86	3.36	3.782 (6)	113
N4—H4⋯S1^iv^	0.86	3.38	3.818 (6)	114

Symmetry codes: (ii) 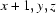; (iii) 
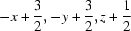
; (iv) 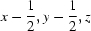.

**Fig. 1.**
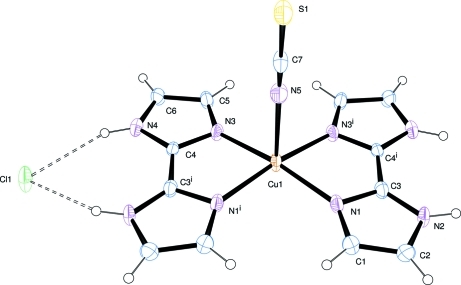
A view of the structure of (I) with displacement ellipsoids drawn at the 30% probability level. Symmetrical code (i) 2 - x, y, z

**Fig. 2.**
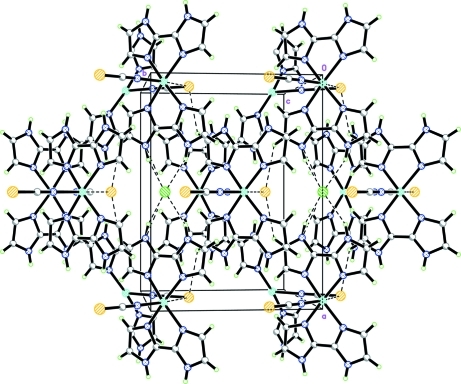
The packing view in (I). Cu (Light cyan); Cl (green); S (yellow); N (blue); C (gray)

## supplementary crystallographic information

### Comment

The neutral molecule 2,2'-biimidazole (H_2_biim) and its monoanionic derivative(Hbiim^-^) is a particular organic target for construction of hybrid materials. Its molecular moieties possess a double property, namely they can be coordinated to metal centres and can act as a donor in hydrogen bonding interactions (Tadokoro *et al.*, 2000). The thiocyanate is a versatile briding ligand (Ribas *et al.*, 1998), we think that the self-assembly of these ligand with metal ions should yield structure fascinating compounds. Thus, the title compound (I) was synthesized and its crystal structure is reported here.

The X-ray crystallographic analysis shows that the crystal structure of (I) consists of [Cu(NCS)(biim)_2_]^+^ cation and Cl^-^ anions(Fig 1). Cu(II) ion adopts a five coordinated square pyramidal geometry consisting of a nitrogen atom(N5) from thiocyanato anion and four nitrogen atoms (N1, N1^i^, N3 and N3^i^) from two coordinating biimidazole ligands. Four nitrogen atoms of two chelating H_2_biim ligands form the basal plane of the pyramid and the apical position is occupied by the thiocyanate ligand which is coordinated in the axial position through the nitrogen atom. Bond distances of the Cu—N1 and Cu—N3(biim) [2.014 (6) and 2.031 (5) Å](Table 1) are shorter than the apical Cu1—N5(SCN^-^) distance[2.344 (10) Å]. These distances lie in the range reported for biimidazole-Cu complexes (Govor *et al.*,2008). The chelating H_2_biim ligands are almost planar and dihedral angle of two biimidazole plane is 6.32°. Meanwhile, In the crystal,molecules are linked by hydrogen bond interaction (N—H···Cl) forming the three-dimensional architecture(Fig 2).

### Experimental

All chemicals were of reagent grade, were commercially available and were used without further purification. CuCl (0.099 g, 0.10 mmol) dissloved in 10.0 ml of ethanol solution was added to 10.0 ml of ethanol solution of H_2_biim (0.0134 g, 0.10 mmol) with stirring. Half an hour later, KSCN (0.0195 g, 0.20 mmol) was slowly added above the mixture. Black green crystal of [Cu(biim)_2_(SCN)]Cl were separated from the mother liquor by slow evaporation at room temperature after two weeks.

### Refinement

H atoms attached to C and *N*(biimidazole) atoms of (I) were placed in geometrically idealized positions with C*sp*^2^—H = 0.93Å and N—H=0.86Å and constrained to ride on their parent atoms.

### Crystal data

**Table:**

[Cu(NCS)(C_6_H_6_N_4_)_2_]Cl	*F*(000) = 860
*M**_r_* = 425.37	*D*_x_ = 1.800 Mg m^−^^3^
Orthorhombic, *C**m**c*2_1_	Mo *K*α radiation, λ = 0.71073 Å
Hall symbol: C 2c -2	Cell parameters from 4272 reflections
*a* = 12.900 (5) Å	θ = 2.1–26.6°
*b* = 9.442 (4) Å	µ = 1.71 mm^−^^1^
*c* = 12.888 (5) Å	*T* = 298 K
*V* = 1569.8 (11) Å^3^	Block, green
*Z* = 4	0.10 × 0.10 × 0.10 mm

### Data collection

**Table:**

Bruker SMART CCD area-detector diffractometer	1291 independent reflections
Radiation source: fine-focus sealed tube	1254 reflections with *I* > 2σ(*I*)
graphite	*R*_int_ = 0.046
ω scans	θ_max_ = 25.0°, θ_min_ = 3.1°
Absorption correction: multi-scan (*SADABS*; Sheldrick, 1996)	*h* = −14→15
*T*_min_ = 0.610, *T*_max_ = 0.847	*k* = −11→10
3518 measured reflections	*l* = −13→15

### Refinement

**Table:**

Refinement on *F*^2^	Secondary atom site location: difference Fourier map
Least-squares matrix: full	Hydrogen site location: inferred from neighbouring sites
*R*[*F*^2^ > 2σ(*F*^2^)] = 0.047	H-atom parameters constrained
*wR*(*F*^2^) = 0.096	*w* = 1/[σ^2^(*F*_o_^2^) + (0.0005*P*)^2^ + 5.3473*P*] where *P* = (*F*_o_^2^ + 2*F*_c_^2^)/3
*S* = 1.20	(Δ/σ)_max_ < 0.001
1291 reflections	Δρ_max_ = 0.45 e Å^−^^3^
121 parameters	Δρ_min_ = −0.45 e Å^−^^3^
1 restraint	Absolute structure: Flack (1983), 539 Friedel pairs
Primary atom site location: structure-invariant direct methods	Flack parameter: 0.06 (3)

### Special details

**Table:**

Geometry. All e.s.d.'s (except the e.s.d. in the dihedral angle between two l.s. planes) are estimated using the full covariance matrix. The cell e.s.d.'s are taken into account individually in the estimation of e.s.d.'s in distances, angles and torsion angles; correlations between e.s.d.'s in cell parameters are only used when they are defined by crystal symmetry. An approximate (isotropic) treatment of cell e.s.d.'s is used for estimating e.s.d.'s involving l.s. planes.
Refinement. Refinement of *F*^2^ against ALL reflections. The weighted *R*-factor *wR* and goodness of fit *S* are based on *F*^2^, conventional *R*-factors *R* are based on *F*, with *F* set to zero for negative *F*^2^. The threshold expression of *F*^2^ > σ(*F*^2^) is used only for calculating *R*-factors(gt) *etc*. and is not relevant to the choice of reflections for refinement. *R*-factors based on *F*^2^ are statistically about twice as large as those based on *F*, and *R*- factors based on ALL data will be even larger.

### Fractional atomic coordinates and isotropic or equivalent isotropic displacement parameters (Å^2^)

**Table:**

	*x*	*y*	*z*	*U*_iso_*/*U*_eq_	
Cu1	0.5000	0.39548 (11)	0.38462 (9)	0.0307 (3)	
S1	0.5000	0.7886 (3)	0.1136 (2)	0.0476 (7)	
N1	0.6167 (3)	0.4872 (6)	0.4638 (4)	0.0317 (12)	
N2	0.7857 (4)	0.4990 (6)	0.4874 (4)	0.0316 (13)	
H2A	0.8509	0.4821	0.4808	0.038*	
N3	0.3808 (4)	0.2803 (5)	0.3256 (4)	0.0294 (12)	
N4	0.2133 (4)	0.2519 (6)	0.3172 (4)	0.0333 (13)	
H4	0.1484	0.2647	0.3294	0.040*	
N5	0.5000	0.5634 (10)	0.2506 (7)	0.044 (2)	
C1	0.6381 (5)	0.5905 (6)	0.5367 (5)	0.0321 (15)	
H1	0.5893	0.6466	0.5703	0.039*	
C2	0.7418 (5)	0.5963 (8)	0.5509 (6)	0.0369 (18)	
H2	0.7767	0.6563	0.5962	0.044*	
C3	0.7087 (4)	0.4344 (7)	0.4372 (5)	0.0302 (15)	
C4	0.2915 (4)	0.3246 (6)	0.3605 (4)	0.0248 (14)	
C5	0.3575 (5)	0.1711 (8)	0.2579 (5)	0.0359 (16)	
H5	0.4062	0.1168	0.2225	0.043*	
C6	0.2537 (5)	0.1550 (10)	0.2509 (6)	0.0345 (18)	
H6	0.2176	0.0911	0.2094	0.041*	
C7	0.5000	0.6548 (10)	0.1940 (8)	0.034 (2)	
Cl1	0.0000	0.3818 (3)	0.4227 (2)	0.0501 (8)	

### Atomic displacement parameters (Å^2^)

**Table:**

	*U*^11^	*U*^22^	*U*^33^	*U*^12^	*U*^13^	*U*^23^
Cu1	0.0153 (5)	0.0344 (6)	0.0425 (6)	0.000	0.000	−0.0068 (6)
S1	0.0303 (13)	0.0513 (17)	0.0612 (19)	0.000	0.000	0.0124 (14)
N1	0.019 (2)	0.039 (3)	0.037 (3)	0.004 (2)	0.004 (2)	0.007 (3)
N2	0.015 (2)	0.033 (3)	0.047 (4)	0.001 (2)	−0.002 (2)	−0.004 (3)
N3	0.019 (2)	0.033 (3)	0.036 (3)	−0.005 (2)	−0.002 (2)	−0.005 (2)
N4	0.021 (3)	0.037 (3)	0.042 (3)	−0.001 (2)	−0.005 (2)	0.002 (3)
N5	0.031 (4)	0.055 (6)	0.046 (6)	0.000	0.000	−0.006 (4)
C1	0.032 (3)	0.025 (4)	0.040 (4)	−0.003 (3)	0.003 (3)	−0.004 (3)
C2	0.037 (4)	0.041 (5)	0.033 (4)	−0.005 (3)	−0.001 (3)	−0.002 (4)
C3	0.021 (3)	0.035 (4)	0.035 (4)	0.003 (3)	0.002 (3)	0.003 (3)
C4	0.018 (3)	0.032 (3)	0.024 (4)	−0.003 (2)	−0.002 (2)	0.010 (3)
C5	0.022 (3)	0.043 (4)	0.042 (4)	0.001 (3)	0.005 (3)	−0.001 (3)
C6	0.026 (4)	0.039 (4)	0.038 (4)	−0.010 (3)	0.001 (3)	−0.004 (3)
C7	0.021 (4)	0.026 (5)	0.054 (7)	0.000	0.000	0.006 (5)
Cl1	0.0206 (11)	0.0479 (15)	0.082 (2)	0.000	0.000	−0.0179 (14)

### Geometric parameters (Å, °)

**Table:**

Cu1—N1	2.014 (6)	N4—C4	1.342 (7)
Cu1—N1^i^	2.014 (6)	N4—C6	1.356 (10)
Cu1—N3	2.031 (5)	N4—H4	0.8600
Cu1—N3^i^	2.031 (5)	N5—C7	1.130 (13)
Cu1—N5	2.344 (10)	C1—C2	1.351 (9)
S1—C7	1.634 (11)	C1—H1	0.9300
N1—C3	1.332 (7)	C2—H2	0.9300
N1—C1	1.382 (8)	C3—C4^i^	1.432 (8)
N2—C3	1.333 (8)	C4—C3^i^	1.432 (8)
N2—C2	1.354 (9)	C5—C6	1.352 (9)
N2—H2A	0.8600	C5—H5	0.9300
N3—C4	1.305 (7)	C6—H6	0.9300
N3—C5	1.383 (8)		
			
N1—Cu1—N1^i^	96.7 (3)	C6—N4—H4	125.7
N1—Cu1—N3	170.3 (2)	C7—N5—Cu1	172.8 (8)
N1^i^—Cu1—N3	81.62 (18)	C2—C1—N1	108.6 (6)
N1—Cu1—N3^i^	81.62 (18)	C2—C1—H1	125.7
N1^i^—Cu1—N3^i^	170.3 (2)	N1—C1—H1	125.7
N3—Cu1—N3^i^	98.4 (3)	C1—C2—N2	107.8 (7)
N1—Cu1—N5	94.7 (2)	C1—C2—H2	126.1
N1^i^—Cu1—N5	94.7 (2)	N2—C2—H2	126.1
N3—Cu1—N5	95.0 (2)	N1—C3—N2	111.6 (6)
N3^i^—Cu1—N5	95.0 (2)	N1—C3—C4^i^	116.5 (6)
C3—N1—C1	105.1 (5)	N2—C3—C4^i^	131.9 (6)
C3—N1—Cu1	112.0 (5)	N3—C4—N4	110.9 (5)
C1—N1—Cu1	142.9 (4)	N3—C4—C3^i^	118.2 (5)
C3—N2—C2	106.9 (5)	N4—C4—C3^i^	131.0 (6)
C3—N2—H2A	126.5	C6—C5—N3	110.0 (7)
C2—N2—H2A	126.5	C6—C5—H5	125.0
C4—N3—C5	105.3 (5)	N3—C5—H5	125.0
C4—N3—Cu1	111.5 (4)	C5—C6—N4	105.2 (7)
C5—N3—Cu1	143.1 (4)	C5—C6—H6	127.4
C4—N4—C6	108.6 (5)	N4—C6—H6	127.4
C4—N4—H4	125.7	N5—C7—S1	179.1 (10)
			
N1^i^—Cu1—N1—C3	172.7 (3)	C1—N1—C3—N2	−0.8 (7)
N3^i^—Cu1—N1—C3	2.3 (4)	Cu1—N1—C3—N2	177.9 (4)
N5—Cu1—N1—C3	−92.0 (5)	C1—N1—C3—C4^i^	−179.4 (5)
N1^i^—Cu1—N1—C1	−9.4 (9)	Cu1—N1—C3—C4^i^	−0.6 (7)
N3^i^—Cu1—N1—C1	−179.7 (7)	C2—N2—C3—N1	1.1 (8)
N5—Cu1—N1—C1	85.9 (7)	C2—N2—C3—C4^i^	179.4 (6)
N1^i^—Cu1—N3—C4	3.8 (4)	C5—N3—C4—N4	−1.3 (7)
N3^i^—Cu1—N3—C4	173.9 (3)	Cu1—N3—C4—N4	177.1 (4)
N5—Cu1—N3—C4	−90.3 (4)	C5—N3—C4—C3^i^	177.0 (5)
N1^i^—Cu1—N3—C5	−178.8 (8)	Cu1—N3—C4—C3^i^	−4.6 (6)
N3^i^—Cu1—N3—C5	−8.6 (9)	C6—N4—C4—N3	0.2 (8)
N5—Cu1—N3—C5	87.1 (7)	C6—N4—C4—C3^i^	−177.8 (7)
C3—N1—C1—C2	0.2 (7)	C4—N3—C5—C6	2.0 (8)
Cu1—N1—C1—C2	−177.8 (6)	Cu1—N3—C5—C6	−175.5 (6)
N1—C1—C2—N2	0.5 (8)	N3—C5—C6—N4	−1.9 (9)
C3—N2—C2—C1	−1.0 (8)	C4—N4—C6—C5	1.1 (9)

Symmetry codes: (i) −*x*+1, *y*, *z*.

### Hydrogen-bond geometry (Å, °)

**Table:**

*D*—H···*A*	*D*—H	H···*A*	*D*···*A*	*D*—H···*A*
N2—H2A···Cl1^ii^	0.86	2.27	3.092 (5)	160
N4—H4···Cl1	0.86	2.52	3.305 (6)	153
N2—H2A···S1^iii^	0.86	3.36	3.782 (6)	113
N4—H4···S1^iv^	0.86	3.38	3.818 (6)	114

Symmetry codes: (ii) *x*+1, *y*, *z*; (iii) −*x*+3/2, −*y*+3/2, *z*+1/2; (iv) *x*−1/2, *y*−1/2, *z*.
